# Treatment of Cancer Pain by Targeting Cytokines

**DOI:** 10.1155/2015/984570

**Published:** 2015-10-11

**Authors:** I. Vendrell, D. Macedo, I. Alho, M. R. Dionísio, L. Costa

**Affiliations:** ^1^Medical Oncology Department, Santa Maria Hospital, Northern Lisbon Hospital Center, Avenida Professor Egas Moniz, 1649-035 Lisbon, Portugal; ^2^Costa Lab, Instituto de Medicina Molecular, Faculdade de Medicina da Universidade de Lisboa, Avenida Professor Egas Moniz, 1649-035 Lisboa, Portugal

## Abstract

Inflammation is one of the most important causes of the majority of cancer symptoms, including pain, fatigue, cachexia, and anorexia. Cancer pain affects 17 million people worldwide and can be caused by different mediators which act in primary efferent neurons directly or indirectly. Cytokines can be aberrantly produced by cancer and immune system cells and are of particular relevance in pain. Currently, there are very few strategies to control the release of cytokines that seems to be related to cancer pain. Nevertheless, in some cases, targeted drugs are available and in use for other diseases. In this paper, we aim to review the importance of cytokines in cancer pain and targeted strategies that can have an impact on controlling this symptom.

## 1. Introduction

Pain is a common symptom of inflammatory-related conditions. In cancer, pain is more frequent in advanced stages with great impact on the quality of life. Cancer pain affects 17 million people worldwide, and its prevalence ranges from 33% in patients after curative treatment to 64% in the metastatic setting and 75–90% will experience moderate to severe pain [1].

Pain can be caused by several different mechanisms, usually by compression, ischemia, and invasion of structures such as nerves. Inflammation is one of the causes of the majority of cancer symptoms, including pain, fatigue, cachexia, and anorexia [2]. Nevertheless, the actual etiology of cancer pain is to this day unknown and cancer seems to generate a specific neurochemical pain state, distinct from inflammatory and neuropathic pain [3, 4].

One of the most accepted hypothesis is that, within a malignant tumor, not only cancer cells but also lymphocytes, fibroblasts, endothelial cells, and neurons produce mediators that are released in the microenvironment and might activate nociceptors [3].

Among the released substances are neurotrophic factors [5], endothelin [6], and formaldehyde [7]. Cytokines are also potential mediators produced by cancer cells and cells recruited to the microenvironment.

Cytokines are small (5 kDa–140 kDa) released secreted proteins by the cells in response to different stimuli that have a specific effect on the interactions and communications between cells [8]. Interferons were the first cytokines discovered in 1957 and soon after the concept was expanded to include chemokines, interleukins, lymphokines, and tumor necrosis factor. The release of different cytokines in a controlled sequence is responsible for the production of different final mediators involved in the induction of inflammatory signs. They are released primarily by antigen presenting cells and can have both pro- and anti-inflammatory functions that will vary according to the microenvironment [9, 10]. They can act on the cells that secrete them, autocrine action, on nearby cells, paracrine action, or, sometimes, on distant cells [11, 12].

The first cytokines described as participating in the development of inflammatory pain were interleukin-1*β* (IL-1*β*), tumor necrosis factor *α* (TNF-*α*), and IL-6. These cytokines are considered proinflammatory [11].

Cancer pain can be caused by different mediators: immune system cells infiltrating the tumor: neutrophils, T cells, and macrophages secrete prostaglandins, endothelin, TNF-*α*, Transforming Growth Factor (TGF), IL-1 and IL-6, Epidermal Growth Factor (EGF), and Platelet Derived Growth Factor (PDGF) [1, 9]. These mediators act in primary efferent neurons directly or indirectly, causing pain.

Cytokines have an additional role in pain. It has been suggested that the plasma concentrations of cytokines such as IL-8, IL-12 (p40 and p70), eotaxin, and macrophage inflammatory protein- (MIP-) 1*α* and MIP-1*β* are associated with the effectivity of morphine treatment. In one study, patients resistant to morphine treatment had lower concentrations of these plasma cytokines [13]. Although the underlying mechanism is still unknown, it is possible that this results from a cross talk between cytokine signaling and opioid receptor signaling: on one hand, the activation of chemokine receptor 1 (CCR1) by cytokines results in the internalization of *μ* opioid receptors and inhibition of their function [14]. On the other hand, the prolonged activation of opioid receptors might itself inhibit the function of chemokine receptors on leukocytes [15].

Spinal proinflammatory cytokines are known pain-enhancing signals. In animal models, chronic intrathecal opioid administration induces activation of spinal cord glia and also the release of spinal IL-1*β* which are mechanisms implicated in the development of hyperalgesia, allodynia, and analgesic tolerance [16]. The administration of morphine to lumbar spinal dorsal cord cells causes an increase in the release of proinflammatory cytokines and chemokines which is detectable in less than 5 minutes after the exposure [14]. This favors the hypothesis that proinflammatory cytokines are correlated with opioid tolerance and opioid induced hyperalgesia [14].

Moreover, variants in genes encoding for cytokines have been suggested as candidates of risk of a variety of cancers and both genetic and nongenetic factors are relevant in the severity of pain. For instance, considering persistent breast pain following breast cancer surgery, there are known associations between previously identified extreme persistent breast pain phenotypes (i.e., no pain versus severe pain) and single nucleotide polymorphisms (SNPs) that suggest a role for cytokine gene polymorphisms [17]. In non-small-cell lung cancer, there also seems to be an influence of SNP in IL-8 and severe pain in white patients [18].

**Figure 1:**
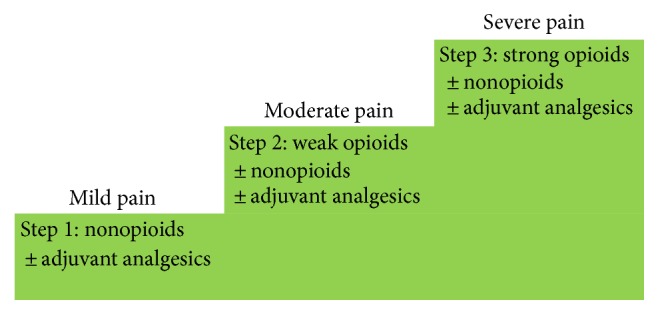
WHO's cancer pain relief ladder for adults.

Currently, the treatment of cancer pain relies mostly on the severity and type of pain and does not include cytokine targeted treatments. In fact, the World Health Organization (WHO) proposes a model for the treatment of cancer pain that uses severity as the main modulator of analgesic treatment. It includes both opioid and nonopioid drugs (Figure 1).

Nonetheless, cancer pain treatment has different options targeting inflammation. Nonsteroid anti-inflammatory drugs (NSAIDs) and corticosteroids [19] are widely used examples of such drugs. Statins might also be helpful in controlling this symptom, by acting as anti-inflammatory agents [20]. Several other agents have been reported, although their actual efficacy remains to be proven (Table 1).

Knowing the mechanisms behind cancer pain and consequently how to specifically target it with the minimal side effects will help physicians to manage this symptom and will consequently have great impact on quality of life.

## 2. General Mechanisms of Cancer Pain

One of the most challenging features in treating cancer pain is understanding the underlying mechanisms. The use of animal models helped clarifying the molecular, biochemical, and neurobiological pathways that are involved in cancer pain. There are several cancer pain experimental models currently used to understand these mechanisms [21]. The first models were mice with primary or metastatic bone cancer [1]. It has been observed that in this case a nonpainful level of mechanical stress can induce the release of substance P from primary afferent fibers that terminate in the spinal cord. Substance P, in turn, binds to and activates the neurokinin-1 receptor and also induces expression of the transcription factor c-Fos on spinal cord neurons [22]. Usually, this condition only occurs with noxious stimuli. Therefore, peripheral sensitization of nociceptors is involved in the generation and maintenance of bone cancer pain [1, 23].

The nociceptor stimulation by tumors occurs due to the release of various factors by immune cells that constitute the tumor. The factors that sensitize or directly excite primary afferent neurons include prostaglandins, TNF-*α*, endothelins, IL-1 and IL-6, EGF, TGF-*β*, and PDGF [9]. Their receptors are expressed by primary afferent neurons [1]. Prostaglandins and endothelins are also thought to be involved in the regulation of angiogenesis and tumor growth and can therefore be important in reducing tumor growth and metastasis [24].

There are other characteristics related to the tumor that contribute to cancer pain such as tumor-induced acidosis [1]. The intra- and extracellular environment of solid tumors is characterized by lower pH. It is known that local acidosis is a hallmark of tissue injury and induces excitation of sensory neurons. The acidic microenvironment adjacent to the richly innervated periosteum is a mechanism associated with pain in metastatic bone cancer [25]. Tumor-induced release of protons and acidosis might be particularly important in the generation of bone cancer pain [26]. The osteoclasts reabsorb bone by maintaining an extracellular microenvironment of low pH at the osteoblast-mineralized bone interface. Most sensory neurons that innervate bone express transient receptor potential vanilloid-1 (TRPV1) and/or acid-sensing ion channels (ASICs) [26]. The TRPV1 is a Ca2+ permeable ionotropic receptor and his antagonism in a mouse bone cancer pain model attenuates the movement-evoked nociceptive behaviors. (B) These findings contribute to the unveiling of the etiology of bone cancer pain, with no target drugs already in practice. However, this effect was already seen in a soft tissue cancer model with the injection of SCC [27].

Despite the fact that tumors are not highly innervated by sensory neurons, their rapid growth can cause mechanical injury, compression, and ischemia, producing pain. The proteolytic activity is critical to carcinogenesis and cancer pain. Cancer associated trypsin has been identified in cancers such as ovarian carcinoma, pancreatic cancer, hepatocellular and cholangiocarcinomas, lung neoplasms, colorectal cancers, fibrosarcoma, gastric cancer, and oral cancer (D). Proteases activate cell surface receptors on primary afferent nociceptors within cancer microenvironment, either directly or via peptide products. Proteolytic enzymes that are produced by the tumor cells can also cause injury to sensory and sympathetic fibers, causing neuropathic pain [1]. Protease activated receptors (PARs) are activated by proteolytic (enzymes) cleavage or by a short peptides ligand with similar sequence. There is release of substance P and calcitonin gene related protein from C-fibers in peripheral tissues with multiple second messenger pathways activated, like TRPV1-dependent thermal and TRPV4-dependent mechanical hyperalgesia [28, 29].

Animal studies demonstrated that cancer pain is in part maintained by a state of central sensitization in which there is an increased transmission of nociceptive information, promoted by neurochemical changes (such as increased levels of glutamate and other excitatory neurotransmitters, with astrocyte hypertrophy) in the spinal cord and forebrain. The upregulation of the prohyperalgesic peptide dynorphin was observed in these models [1].

Additionally, not only is cancer pain transmitted by the classically described spinothalamic tract, but also there is evidence that other pathways such as those in the dorsal column might be implicated [1]. As cancer pain, more specifically visceral pain, is frequently bilateral, it might be difficult to manage by surgical approaches since bilateral cordotomies can have relevant complications [1].

## 3. The Role of Cytokines in Cancer Pain

**Figure 2:**
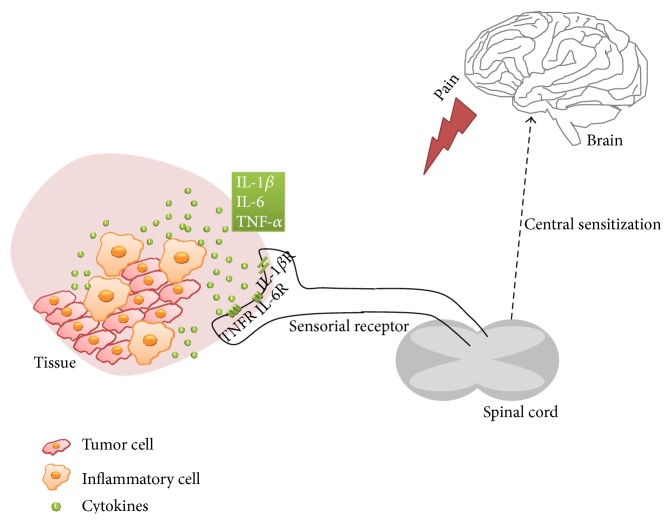
Cytokines in cancer pain. The tumor is composed not only by cancer cells but also by inflammatory cells, among others. The inflammatory cells present in tumor tissue release cytokines that sensitize the sensorial receptor. The painful stimuli, mediated by cytokines, are detected by the sensorial receptor and are transmitted to neurons in spinal cord. The signal is then transmitted to the brain. Adapted from Molecular Mechanisms of Cancer Pain, Nature Reviews Cancer.

It is well accepted that cytokines constitute a link between cellular injuries or immunological recognition and the local or systemic signs of inflammation [12]. In this way, cytokine activation and dysregulation are implied in a variety of inflammatory diseases and also in cancer [30]. In fact, they are produced at high levels in cancer with effects that are directly neuronal or immunological. (A) Actually, there is evidence that these mediators are involved in the initiation and persistence of pathological pain by direct and indirect action on nociceptive sensory neurons.  Figure 2 schematizes the role of cytokines in cancer pain. Some cytokines are also related to nerve injury/inflammation-induced central sensitization and are associated with hyperalgesia and allodynia phenomena [11, 30]. It has been demonstrated that inflammatory stimuli do not directly induce the release of prostaglandins and sympathetic amines. Instead, a well-defined sequential cytokine cascade precedes the release of these final hypernociceptive mediators. This cytokine cascade is modulated by a parallel release of anti-inflammatory cytokines [12].

Proinflammatory cytokines are produced by activated macrophages. There is a strong evidence of their association with pathological pain, especially IL-1*β*, IL-6, and TNF-*α* [11, 12, 30].

Production and secretion of IL-1*β* are associated with pain in pathological conditions like tumor growth. It is released by monocytes and macrophages and by nonimmune cells, during processes of cell injury, infection, invasion, and inflammation. It is expressed in nociceptive dorsal root ganglion (DRG) neurons and in Schwann cells on peripheral nerves and plays a central role in the generation of hyperalgesia [11]. IL-1*β* was the first cytokine reported to mediate inflammatory nociception in experimental animals [1]. Corroborating these findings, IL-1*β* was demonstrated to stimulate the expression of cyclooxygenase 2 (COX-2) and the subsequent release of its products, prostaglandins [11, 12]. Indeed, in some studies, small doses of IL-1*β* produce a severe mechanical hypernociception that depends on the release of prostanoids [30]. This situation can be prevented by experimental administration of endogenous IL-1 receptor antagonist (IL-1ra) or a COX-2 inhibitor, suggesting that induced hypernociception is mediated by the activation of specific membrane receptors and the subsequent synthesis of prostaglandins [12]. Hence, the peripheral pronociceptive action of IL-1*β* is mediated by a complex signaling cascade. Besides the sensitization of nociceptor by prostanoids, direct nociceptor activation by inflammatory mediators such as serotonin, histamine, or ATP might occur in models of inflammatory pain. In these models, the participation of IL-1*β* was also demonstrated [31–33]. In addition, there is evidence that IL-1*β* participates with other cytokines in the genesis of neuropathic pain: it was observed that chronic constriction injury of peripheral nerves induces lumbar spinal IL-1*β* expression. Similarly, IL-1*β* or IL-1R1 gene disruption also impairs the neuropathic hypernociception, while transgenic overexpressing IL-1R1 mice have increased neuropathic pain [12].

The serum levels of pleiotropic cytokine IL-6 are increased in malignant tumors. It is markedly upregulated during various pathological situations and has both anti- and proinflammatory effects. IL-6 is generally associated with hyperalgesia and has been shown to play a central role in neuronal reaction to nerve injury. Suppression of IL-6 receptor (IL-6R)* in vivo* by application of anti-IL-6R antibodies led to reduced regenerative effects. IL-6 is also involved in microglial and astrocytic activation and in regulation of neuronal neuropeptides expression. So, IL-6 contributes to the development of neuropathic pain behavior following a peripheral injury [11]. Some data suggest that the inflammatory environment determines the capacity of IL-6 to induce IL-1*β* production with the release of prostanoids, causing hypernociception in an animal model [34]. However, when IL-1ra was administered, there was no inhibition of hypernociception [12].

TNF-*α*, also known as cachectin, is considered the prototypic proinflammatory cytokine due to its principal role in initiating the cascade of activation of other cytokines and growth factors in the inflammatory response. After injury or during inflammation, TNF-*α* is synthesized and released by various cell types, mainly monocytes and macrophages. It has also been demonstrated that Schwann cells can produce TNF-*α* after injury, suggesting a possible role in neuropathic pain. Studies with intraplantar injection of TNF-*α* in mice have been shown to induce mechanical allodynia and thermal hyperalgesia. Indeed, subcutaneously injected TNF-*α* lowers mechanical activation threshold in C nociceptors in the sural nerves of mice [12]. These data demonstrated that blocking TNF-*α* reduces hyperalgesia in models of painful neuropathy. It interacts with target cells through high-affinity membrane receptors: tumor necrosis factor receptor 1 (TNFR1) and tumor necrosis factor receptor type 2 (TNFR2). The effects associated with experimental hyperalgesia have been shown to be dependent on TNFR1 following experimental nerve lesion. Downstream of TNFR activation hyperalgesia induced by inflammation or nerve injury is mediated via p38 MAPK. But TNF-*α* also acts by nuclear factor kB (NF-kB) activation of inflammation and by stress-activated protein kinases (SPAKs) [11, 12]. The TNF-*α* hypernociceptive effect was partially inhibited by COX inhibitor and *β* adrenergic receptor antagonists (like atenolol) and eliminated by cotreatment with these drugs, suggesting that this function is mediated by prostanoids and sympathetic amines [12]. In fact, it is known that TNF-*α* induces the production of IL-1*β*, IL-6, and IL-8. The cytokine cascade results in the activation of COX-2 dependent prostanoid release and release of catecholamines from sympathetic fibers [35]. On the other hand, TNF-*α* also participates in bone destruction, which is a major source of tumor related pain. TNF-*α* can regulate differentiation and activation of osteoclasts by its receptor, independent of complex receptor activator of NF-kB (RANK) and RANK ligand (RANKL) [12]. Moreover, there is evidence that the latter and TNF-*α* cooperate in osteoclastogenesis [35]. Despite the evidences described above that the hypernociceptive effects of cytokines are indirect, it has been reported that sensory neurons express TNF-*α* and IL-1*β* receptors, suggesting that these cytokines might also directly sensitize the nociceptor during inflammation. It was demonstrated that TNF-*α* evokes action potentials in nociceptive neurons when applied topically to peripheral axons* in vivo* and increases the sensitivity to mechanical and chemical stimuli. On the other hand, IL-1*β* acts on sensory neurons increasing their susceptibility to noxious heat.

## 4. The Role of Cytokines in Chemotherapy Induced Pain

Cancer therapies such as cytotoxic agents can, themselves, cause pain in cancer patients. They usually generate neuropathic pain [36]. Chemotherapeutic agents that are more often associated with neuropathy are not only platinum derived agents (oxaliplatin, cisplatin, and carboplatin) but also taxanes (paclitaxel and docetaxel), vincristine, and others such as bortezomib, thalidomide, lenalidomide, and epothilones [36].

Pain secondary to chemotherapy is probably due to multiple mechanisms. Most chemotherapy agents are able to penetrate the blood-nerve-barrier and bind to the dorsal root ganglia and peripheral axons thus increasing their potential neurotoxicity [36].

Some chemotherapeutic agents have the ability to disrupt tubulin function. Tubulin polymerization is necessary for axonal transport of trophic factors, and drugs that interfere with this process can cause degeneration of sensory neurons and release of proinflammatory cytokines that directly sensitize primary afferent nociceptors [1].

Likewise, cytotoxic agents might also induce neuropathic pain by activating ion channels in the plasma membrane on dorsal root ganglia and dorsal horn neurons. The inflammatory process that is triggered in the immune system cells by chemotherapeutic agents, with the subsequent release of proinflammatory cytokines, can also trigger changes in the sensory neurons and ultimately alter nociceptive processing [36, 37].

Although different chemotherapeutic agents generate neurotoxicity by different mechanisms, overall proinflammatory cytokines not only can contribute, in fact, to axonal damage by activating the inflammatory process but also can modulate spontaneous nociceptor sensitivity and activity, a mechanism involved in allodynia and hyperalgesia after nerve injury [36].

## 5. Chemokines

Another set of molecules with particular relevance in pain development is chemokines. Their most important role is the recruitment of white blood cells to the site of inflammation, but they also play other parts such as promoting angiogenesis and modulating the immune response and they are involved in fever as well.

There are approximately 50 chemokine ligands, which can be divided into four families based on the sequences of their cysteine residues: CC-chemokine ligands (CCL), CXC-chemokine ligands, CX_3_C-chemokine ligands, and XC-chemokine ligands [38]. Chemokine receptors are widely spread in white blood cells, neurons, and glial cells [39]. The relationship between chemokine ligands and their receptors is complicated, due to the fact that each chemokine ligand has multiple receptors. Since some chemokine receptors, such as CCR2, CCR5, CXCR4, and CX3CR1, are located in primary afferent neurons or secondary neurons of the DRG, chemokines (such as CCL2 and CCL3) can potentially alter pain transmission and produce pain behaviors [40]. On the other hand, some of these chemokines, such as CCL2, participate in pain regulation through direct action on sensory neurons and indirect action via leukocyte activation [41]. The chemokine effects on pain sensation are complex, and additional effort is required to clarify the role of these molecules in cancer derived analgesia.

In the complex process of pain in cancer, there are many phenomena difficult to manage. One of the important sensations is the hypernociception. When tissue is damaged, various chemical mediators are released such as prostaglandins, histamine, serotonin, bradykinin, nerve growth factor, cytokines, and chemokines. The chemokines seem to contribute to pain hypersensitivity and spontaneous pain, either by a direct action or by modulation of the activity of nociceptors [42].

The mechanisms underlying the hypernociceptive effects of chemokines remain unclear. Chemokine IL-8/CXCL8 mediates the participation of sympathetic components of the inflammatory hypernociception [11, 12]. IL-8/CXCL8 induces a dose and time-dependent mechanical hypernociception, which is inhibited by *β* adrenergic receptor antagonists, but not by COX inhibitor. There is also evidence that chemokines participate in neuropathic nociceptive response by inducing the monocyte chemoattractant protein-1 (MCP-1) expression in DRG. MCP-1 acts through its receptor CCR2 which is directly involved in neuronal hyperexcitability and neuropathic pain after chronic compression injury [12]. The CX3CL1 chemokine also participates in the pathophysiology of experimental neuropathic pain. It induces microglia IL-1 and IL-6 production, which mediate mechanical and thermal hypernociception [12].

More recently, studies have shown that CXCR3 might be of pivotal importance in bone cancer pain in mice, acting through Akt and extracellular signal-regulated kinase (ERK) signaling pathways [43]. Nevertheless, there are three splicing variants of the CXCR3 receptor, with opposite biological activities still not extensively understood, which limit the use of therapies targeting this axis [44].

Further studies are needed in order to understand the actual relevance of cytokines in cancer pain and eventually develop targeted therapies that will help to provide a better cancer care.

## 6. Targeting Cytokines for the Treatment of Cancer Pain

There are currently several different options targeting inflammation for the treatment of cancer pain (Table 1). Nonsteroid anti-inflammatory drugs (NSAIDs), COX-2 inhibitors, opioids, and corticosteroids [19] are widely used. Several other agents have been reported, although their actual efficacy, in most cases, remains to be proven.

### 6.1. Corticosteroids

Corticosteroids are widely used in cancer treatment and are recommended as adjuvants for cancer pain, as they tend to reduce adverse effects associated with opioid therapy [30, 45]. They exert their anti-inflammatory action through glucocorticoid effects.

Glucocorticoids have two major roles in cancer pain: they reduce the swelling, which is particularly important in central nervous system (CNS) lesions [46] and also reduce the inflammation by diminishing the proinflammatory cytokines.

Corticosteroids might have an effect in different stages of pain (transduction, transmission, modulation, and pain perception) [45]. After synthesis and release resulting from tissue injury, cytokines appear to increase pain perception. By inhibiting the expression of collagenase, responsible for tissue breakdown during inflammatory mechanisms, corticosteroids diminish proinflammatory cytokines and therefore modulate the activation of nociceptive mechanisms [47].

Steroids also seem to have an important role in pain perception at a different level, since there is evidence showing that androgens have analgesic effects in humans while estrogens might have both hyperalgesic and analgesic effects [47, 48].

Steroids bind to specific homodimeric glucocorticoid receptors (GRs) which are expressed in most tissues, forming a complex that will interact with many cytoplasmic molecules and also by direct interaction with DNA sequences known as glucocorticoid response elements (GREs). In particular, NF-*κ*B transcription seems to be antagonized by the GR-ligand complex, by interaction with the p65 subunit of NF-*κ*B, in a protein-protein interaction [49]. Glucocorticoids are reported to have another less important role in repressing NF-*κ*B activity, mainly through the induction of its inhibitor, I*κ*B [49]. Given the important role of NF-*κ*B in activating transcription of proinflammatory cytokines, glucocorticoids will indirectly contribute to the downregulation of cytokines such as TNF-*α* and IL-1*β*, with important anti-inflammatory effects [50].

Despite the rational for administering corticosteroids in cancer patients, several studies do not show a significant improve in analgesia in patients already receiving opioids [51]. There is still a lack of studies and further evidence is still needed. Nevertheless, they seem to have an effect on other symptoms such as fatigue, appetite loss, and patient satisfaction and therefore might continue playing an important role in palliative care [52].

### 6.2. NSAIDs and COX-2 Inhibitors

NSAIDs are commonly used anti-inflammatory agents, recommended as a monotherapy or combined with opioids for improvement of cancer analgesia by increasing pain relief and by reducing opioid dosing and therefore side effects [53]. These drugs act mainly by inhibiting COX-1 and COX-2 and therefore diminishing the conversion of arachidonic acid to prostaglandins and thromboxane. These two isoforms have different roles in pain, as COX-1 is constitutively expressed in most tissues and organs, accounting for most of the adverse effects of NSAIDs, while COX-2 is an inducible enzyme, localized mainly in inflammatory cells and tissues [54]. COX-2 is induced by both mitogens and proinflammatory mediators such as IL-1*β*, IFN-*γ*, and TNF-*α*. COX-2 inhibitors were developed in order to decrease the secondary effects that would result from inhibiting COX-1.

Some NSAIDs can, additionally, inhibit the activation of NF-*κ*B, thus contributing to diminish the production and release of cytokines such as IL-1*β*, TNF-*α*, and IL-6 [55, 56].

Moreover, NSAIDs seem to have another target as they act on hydrogen-gated ion channels directly responsible for lowering the pH causing tissue acidic environment that contributes to pain by sensitizing nociceptors. NSAIDs are also inhibitors of ASIC mRNA transcription (usually induced by inflammation), and these two actions together contribute to NSAID reduction of low-pH induced pain [57].

### 6.3. Opioids

Opioids are the mainstay of cancer pain treatment. Opioid analgesics refer to drugs that include alkaloids extracted from poppy seeds such as morphine and codeine, semisynthetic derivatives of these drugs (oxycodone, hydromorphone, and oxymorphone), synthetic phenylpiperidines (meperidine, fentanyl), and synthetic pseudopiperidines (methadone) [58].

Three main types of opioid receptors were classically described: *μ* opioid receptor (MOP), *δ* opioid receptor (DOP), and *κ* opioid receptor (KOP). More recently, the nociception/orphanin FQ (NOP) has been discovered [58–60].

The analgesia results from the activation of these receptors which will directly inhibit neurons, thus inhibiting spinal cord pain transmission [61]. However, opioids also have diverse immunomodulatory effects through various mechanisms. This was first realized in animal models, when the administration of morphine reduced swelling and peripheral inflammation, an effect not reversed by naloxone administration [62]. These effects seem to result from the influence of opioids on the expression of cell adhesion molecules, which impairs the adhesion of the immune cells to the endothelium [63].

On the other hand, there is also evidence that opioids might interact with TNF-*α* [64]. Data* in vitro* showed that tramadol and ketobemidone and morphine but not fentanyl inhibited the production of TNF and IL-8 mRNA. This was an effect with a significant dose-response relationship (maximum inhibition occurred at millimolar levels) and was not always reversible by naloxone [65]. Therefore, opioids might have a role in the treatment of peripheral inflammatory pain, by interfering with cytokines.

### 6.4. Statins

Statins are well-known cholesterol-lowering agents, with a major role in cardiovascular prevention. They act by inhibiting enzyme 3-hydroxy-3-methylglutaryl-coenzyme A (HMG-CoA) reductase, with reduced mevalonate synthesis and therefore lowering cholesterol levels [20]. Along with this effect, many other substances are decreased such as inflammatory cytokines and proadhesive molecules, therefore also exhibiting anti-inflammatory properties that could be useful for the treatment of cancer pain [66]. Nevertheless, the use of statins in elderly cancer patients resulted in the increase in pain, mainly due to myositis which is thought to be associated with diminished synthesis of Coenzyme Q [67]. Currently, statins have no role in cancer pain control and to our knowledge there are no clinical trials testing this hypothesis.

### 6.5. Anti-TNF Agents

There is solid evidence supporting TNF-*α* as a potential therapeutic target to control inflammatory pain states. Different drugs have been developed such as infliximab (a chimeric anti-TNF-*α* antibody), etanercept (p75 TNF*α* receptor/immunoglobulin G fusion protein which reduces p38 MAPK phosphorylation and allodynia) [11], and adalimumab (human monoclonal anti-TNF-*α* antibody). Despite the data concerning the antinociceptive role of TNF-*α* and the beneficial effect of these drugs on inflammatory diseases (uveitis, psoriasis, and rheumatoid arthritis), there are few studies suggesting their analgesic effect especially in cancer patients [12]. There is a report of two cases concerning the targeted administration of etanercept in an anatomic site proximal to bone metastasis in patients with refractory pain. This leads to rapid, substantial, and prolonged relieve of the complaints [68]. In this case, the improvement might be to the role of TNF-*α* in the vicious cycle of bone metastasis, by inhibiting osteoclast mediated bone reabsorption [68].

Theoretically, anti-TNF agents seem to be natural targeted agents for cancer pain and have in fact been studied for supportive care as TNF seems to be implicated in cachexia, fatigue, and cancer associated depression. Nonetheless, more studies are necessary to validate their analgesic effect, and the risk of opportunistic infections, particularly relevant in cancer patients, should be taken into account while using these immune modulators.

### 6.6. IFN-*γ* Blocking Agents

Interferon gamma (IFN-*γ*) is a dimerized soluble cytokine released along with other proinflammatory cytokines upon stimulation of immune system cells. It has long been reported in leukemia patients that treatment with IFN-*γ* might spontaneously cause pain symptoms [69].

This cytokine appears to be a key modulator of CB2 cannabinoid receptor signaling during neuropathic pain. In response to nerve injury, CB2 receptors modulate glial activation and contribute to the containment of neuropathic pain [70]. IFN-*γ* is a mediator of CB2 signaling and upon release from astrocytes and neurons will contribute to the perpetuation and progression of neuropathic pain [70]. On the other hand, CB1 receptor has different role in neuropathic pain induced by IFN-*γ*. In fact, there are reports showing that despite its high expression in the CNS, disrupting the expression of the CB1 receptor does not seem to have a great impact on the development of neuropathic pain [71]. However, the CB1 receptors expressed in peripheral nociceptors do seem to be involved in this type of pain and its activation actually reduces pain sensitivity [70, 72].

Blocking INF-*γ* is not currently used as a strategy in cancer pain treatment and to our knowledge there are no current trials addressing this question. Similar to anti-TNF agents, by compromising the immune system inhibiting, IFN might carry important risks.

### 6.7. IL-6 Blocking Agents

Given the known increased levels of IL-6 in many malignant tumors and its association with hyperalgesia, targeting IL-6 might have a role in cancer pain [11].

Tocilizumab is a humanized IL-6 receptor monoclonal antibody [73], approved by the FDA and EMA for use in moderately to severely active rheumatoid arthritis, for children with polyarticular juvenile idiopathic arthritis and for the treatment of systemic juvenile idiopathic arthritis (SJIA).

Tocilizumab has been sporadically administered for the treatment of cancer cachexia with success [74, 75]. Nevertheless, there are no reports of its use in cancer pain.

### 6.8. Targeting Chemokines

Chemokines and particularly IL-8/CXCL8 have been implicated in inflammatory hypernociception and are suspected to have a role in perineural invasion associated with pancreatic cancer [11, 76]. Therefore, targeting these molecules might also have a role in the treatment of cancer pain.

A fully humanized anti-IL-8 antibody, ABX-IL8, has been developed and proved to inhibit tumor growth, angiogenesis, and metastasis of human melanoma, when compared with control IgG-treated animals [77]. It was also effective in inhibiting tumor growth in orthotopic bladder xenografts [78]. The preclinical efficacy was never translated into clinical benefit in humans since the clinical trials were abandoned after disappointing results for ABX-IL8 in autoimmune diseases. Therefore, although targeting chemokines in cancer pain might be of use, there is still no available approach for IL-8. Targeting other chemokines or even a combined strategy could be possible approaches.

### 6.9. Endothelin-1 Antagonists

Endothelin-1 (ET-1) is a 21-amino acid peptide with a well-known role in angiogenesis and tumor growth [24]. Several malignant tumors secrete endothelin, such as prostate cancer, pancreatic cancer, colon cancer, ovarian cancer, and renal cell carcinoma [6].

More recently, endothelin has been shown to be implicated in pain, both in humans and animal models [79]. ET-1 is thought to be released by immune system cells, along with cytokines, in response to different stimuli such as tissue damage. Its concentration is variable among different tumors and some malignancies such as oral squamous cell carcinoma produce greater amounts of ET-1 than melanoma. This suggested an important role of the cancer cells as the source of ET-1.

ET-1 is intimately related to cytokines and its implication in pain would occur through ET receptors, believed to be important in inflammatory, neuropathic, and tumoral pain [6]. In fact, regarding cancer pain, ET-1 concentration seems to be more relevant than tumor volume [80].

Selective endothelin receptor antagonists have been tested in animal models; they were able to reduce pain respond and might be an option to explore concerning cancer pain, particularly in tumors known to secrete ET-1 [81].

In clinical trials, zibotentan or atrasentan, two selective ET_A_ receptor antagonists, did not seem to have an impact on overall survival or progression-free survival in patients with hormone refractory prostate cancer [82, 83]. However, atrasentan did seem to have an effect on cancer related bone pain and skeletal related events in prostate cancer patients [82].

### 6.10. Other Agents

Other agents that target inflammation and cytokines are being tested in cancer pain.

Lenalidomide is a second generation analogue of thalidomide, developed in order to maximize the antiangiogenic, antitumorigenic, and immunomodulating activity while reducing side effects such as sedation and neuropathy [84, 85]. It is approved by the FDA and EMA for the treatment of myelodysplastic syndromes and multiple myeloma.

Lenalidomide exerts its anti-inflammatory function by inhibiting production of proinflammatory cytokines such as TNF*α*, IL-1*β*, IL-6, and IL-12 and increasing anti-inflammatory cytokines such as IL-10. It also inhibits the expression of COX-2, therefore diminishing the production of prostaglandins [85].

This drug has been tested in a phase II randomized controlled trial that aimed to access the efficacy and safety of lenalidomide in patients with complex regional pain syndrome type 1. The results showed no increase in toxicity. Yet, there is no difference in pain control as well, not confirming the results of a previous pilot study [86].

Yet, one study by MD Anderson Cancer Center tried to evaluate the efficacy of lenalidomide for the treatment of refractory cancer pain (NCT00684242). Only three patients were recruited from May 2008 to November 2010, with one patient excluded before assignment to groups, and the study was terminated for low accrual. The study aimed to assess the changes in cancer pain from baseline to day 15 using Edmonton Symptom Assessment Scale (ESAS) to measure participant responses to 10 common symptoms (pain, fatigue, nausea, depression, anxiety, drowsiness, shortness of breath, appetite, sleep problems, and feeling of well-being). One participant had no change in pain, while the other had an improvement in two points (symptoms rated on a 0 to 10 scale from 0 “no symptom” to 10 “worst possible symptom”). There is no record of other studies with this drug in cancer pain.

## 7. Conclusion

Inflammation plays an important role in cancer pain and cytokines are key molecules for this process. Although known to be involved in cancer pain, cytokines are seldom targeted. In fact, although already available and in use in other diseases such as rheumatological conditions for their anti-inflammatory properties, most of these drugs have not been tested as analgesics in the setting of cancer pain. Many have not yet been proved relevant in the setting of inflammatory pain. Nevertheless, early therapeutic results, in both animal models and humans, favor the importance of these drugs in cancer pain and warrant further testing.

## Figures and Tables

**Table 1:** Drugs targeting cytokines that have been tested in pain control.

Drug class	Mechanism of action	Cytokines targeted	Indication in cancer pain treatment
Steroids	Inhibit NF-*κ*B	Downregulate TNF-*α* and IL-1	Adjuvants for cancer pain treatment CNS lesions
NSAIDs	COX-1 and COX-2 inhibition	Downregulate IL-1*β*, TNF-*α*, and IL-6.	Adjuvants for cancer pain treatment bone pain
Opioids	Release of endogenous opioid peptides	Possible inhibition of TNF-*α* and IFN-*γ* release	Moderate to severe pain
Statins	Inhibition of HMG-CoA reductase	FGF, EGF, TGF, and PDGF	Not in use
Anti-TNF agents	Multiple mechanisms of TNF blocking (antibody)	TNF	Not in use
IFN-*γ* blocking agents	Interaction with CB2 receptor	IFN-*γ*	Not in use
IL-6 blocking agents	Antibodies	IL-6	Not in use
Targeting chemokines	Antibodies	Chemokines	Not in use
Endothelin antagonists	ET_A_ receptor antagonists	Endothelin	Not in use
